# Sound symbolism processing is lateralized to the right temporal region in the prelinguistic infant brain

**DOI:** 10.1038/s41598-019-49917-0

**Published:** 2019-09-17

**Authors:** Jiale Yang, Michiko Asano, So Kanazawa, Masami K. Yamaguchi, Mutsumi Imai

**Affiliations:** 1 Research and Development Initiative, Chuo University, Tokyo, Japan; 2 Department of Psychology, College of Contemporary Psychology, Rikkyo University, Saitama, Japan; 3 Department of Psychology, Japan Women’s University, Kanagawa, Japan; 4 Department of Psychology, Chuo University, Tokyo, Japan; 5 Faculty of Environment and Information Studies, Keio University, Kanagawa, Japan

**Keywords:** Cognitive ageing, Sensory processing, Human behaviour

## Abstract

Sound symbolism, which is the systematic and non-arbitrary link between a word and its meaning, has been suggested to bootstrap language acquisition in infants. However, it is unclear how sound symbolism is processed in the infants’ brain. To address this issue, we investigated the cortical response in 11-month-old infants in relation to sound-symbolic correspondences using near-infrared spectroscopy (NIRS). Two types of stimuli were presented: a novel visual stimulus (e.g., a round shape) followed by a novel auditory stimulus that either sound-symbolically matched (*moma*) or mismatched (*kipi*) the shape. We found a significant hemodynamic increase in the right temporal area, when the sound and the referent sound were symbolically matched, but this effect was limited to the *moma* stimulus. The anatomical locus corresponds to the right posterior superior temporal sulcus (rSTS), which is thought to process sound symbolism in adults. These findings suggest that prelinguistic infants have the biological basis to detect cross-modal correspondences between word sounds and visual referents.

## Introduction

In traditional linguistics, the arbitrariness of the relationship between sound and meaning is considered a core principle of language^1^. For the majority of words in a lexicon, mapping between sound and meaning may indeed seem arbitrary. However, recent large-scale computational research in which word lists covering nearly two-thirds of the world’s languages were analyzed^2^ found strong associations between speech sound and meanings for some property words (e.g., “small” and i, “full” and p or b) and body part terms (“tongue” and l, “nose” and n). Psychologically, people generally have good sensitivity to sound symbolism, i.e., the non-arbitrary relationships between linguistic sound and meaning^3–6^. For example, the open vowel [a] tends to be associated with large object size while the closed vowel [i] tends to be associated with small object size (i.e., vowel-size symbolism^4^). People judge the nonsense word *maluma* to be better associated with round than angular shapes, while *takete* sounds better for angular shapes (i.e., the *bouba/kiki* effect^5,6^).

The relationship between sound symbolism and children’s language development has been discussed extensively in the recent literature. Sound symbolic words abound in children’s early speech production^7,8^ and in talk directed to infants by caretakers^9^. Previous behavioral studies have also demonstrated that sound symbolism plays a facilitative role in word learning. For example, research has shown that 14-month-old infants benefit from *bouba/kiki* type sound-shape correspondence in an associative word learning task^10^. This scaffolding effect continues into toddlerhood, particularly in verb learning^11–13^.

The idea that sound symbolism scaffolds lexical development^3^ presupposes the ability of infants to detect inherent similarities across sound and other perceptual modalities. A handful of recent behavioral and brain research studies have suggested that prelinguistic infants indeed have such abilities. For example, it has been shown that four-month-old infants detect vowel-size sound symbolism^14^. Some other studies have demonstrated that prelinguistic infants are also sensitive to *bouba/kiki* type sound-shape correspondence^15–17^. Fort, Lammertink, Peperkamp, Guevara-Rukoz, Fikkert, and Tsuji^18^ conducted a meta-analysis of the sound symbolism effect in infants by examining both published and unpublished work, most of which employed behavioral measures. These authors concluded that young infants by and large are sensitive to audition-vision correspondence.

However, understanding of the neural mechanism of sound symbolism remains limited, particularly for infants, but also for adults. Ramachandran and Hubbard^6^ hypothesized that multi-sensory integration at the temporal-parietal-occipital (TPO) junction is critical for sensing sound symbolism. The TPO junction includes the posterior part of the superior temporal sulcus (STS)^19^, which is known to play a key role in the integration of complex featural information such as facial movements and vocal sounds, particularly between audiovisual linguistic signals (reviewed in^20^). Although investigations on the neural mechanism underlying sound symbolism processing are not plentiful even in adults, two previous adult functional magnetic resonance imaging (fMRI) studies that used conventional sound symbolic words in Japanese across different semantic domains have produced results broadly consistent with Ramachandran’s hypothesis in that both identified the involvement of the right STS (superior temporal sulcus) area in sound symbolism processing.

In one study, the auditory presentation of Japanese mimetic words for animal sounds (e.g., *ka-ka*, an onomatopoeia for crow croaks) was found to activate the right STS more strongly than the names of the animals (e.g., *karasu*, the Japanese word for “crow”)^21^. Another study also identified the activation of the right STS area^22^ in two different non-auditory semantic domains, i.e., shape and motion. Regardless of the domain, when the word sound matched the referent, the posterior part of the right STS was activated more strongly than when the word sound and the referent were mismatched. Thus, the previous results suggest that the right STS plays a critical role in processing sound symbolism, serving as a hub to integrate language sound and visual information.

Little is known about how sound symbolism is processed in the infant brain or its ontogenesis. Thus far, only one published study has investigated the brain response to sound symbolism in young infants, which was included in the meta-analysis by Fort *et al*.^15^. Asano and colleagues examined the electroencephalography (EEG) responses of 11-month-old infants when presented with sounds that symbolically matched and mismatched couplings of pseudo-words and visual shapes (e.g., *moma* for round shapes and *kipi* for spikey shapes)^15^. In terms of the event-related potential (ERP) pattern, infants responded differently to the sound-symbolically matching word-shape pairs than mismatching word-shape pairs. The timing and topography were similar to the typical N400 response, which is an index of semantic integration difficulty for both adults^23^ and infants^24,25^. Furthermore, the phase synchronization of the neural oscillations (phase locking value, PLV) increased (as compared with the baseline period) significantly more in the mismatch condition than in the match condition, suggesting that cross-modal binding was achieved quickly in the match condition, but that sustained effort was required in the mismatch condition. An additional brain oscillation analysis showed an increase in the early (<200 ms latency) gamma-band oscillations in the match condition compared with the mismatch condition, which was thought to be related to multisensory integration (reviewed in^26^). Taken together, these results provide some evidence for the hypothesis that infants detect the correspondence between word sounds and referents through spontaneous cross-modal mapping. However, more direct evidence for this hypothesis is warranted: if early sensitivity to sound-shape symbolism in young infants^15–18^ reflects the spontaneous cross-modal mapping ability available before or at the time at which infants start to make conscious efforts in connecting word sounds to their referents, a significant hemodynamic response would be expected in the area corresponding to the right TPO junction area, especially in the area of the STS, where audio-visual information is integrated (adults^27^; 3-month-old infants^28^).

**Figure 1:**
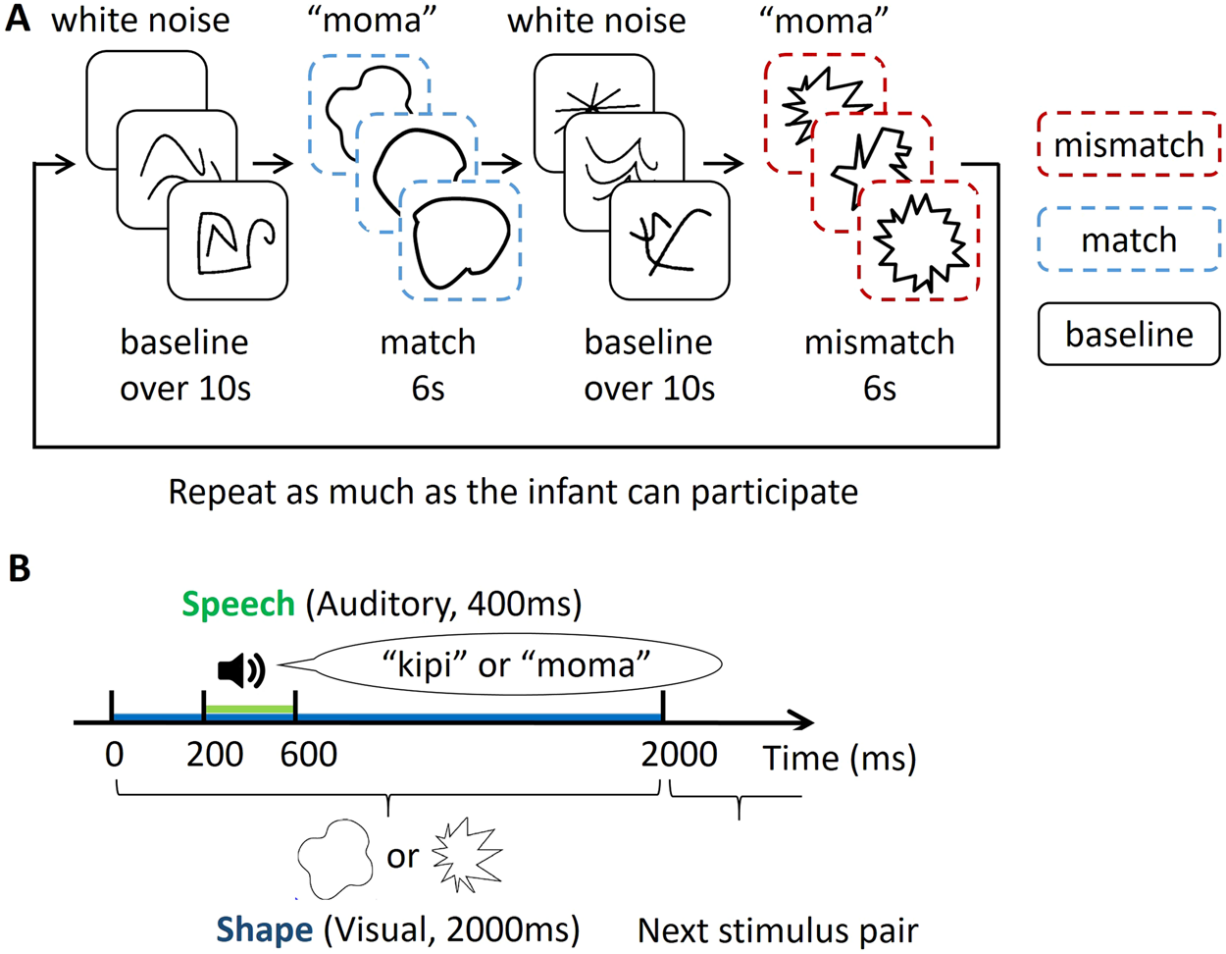
Experimental procedure. (**A**) Schematic of the *moma* condition. The matching and mismatching pairs were presented alternately in a block design. A baseline period, in which neutral shapes were accompanied by white noise, was inserted between the presentation of the matching and mismatching pairs. The experiment continued until the infants could not continue with the task. In each 6-s test block, infants were presented with three different spiky or three different round visual shapes, each followed by the novel word “moma”. The shapes and sounds were sound-symbolically matched (e.g., a round shape followed by “moma”) or mismatched (e.g., a spiky shape followed by “moma”). The duration of the baseline period was over 10 s, and varied across trials as the experimenter started the test trial only when the infant remained focused on the stimuli in the last 2 s. The presentation order of the matched and mismatched pairs was counterbalanced among infants. In the *kipi* condition, the visual stimuli were identical with the *moma* condition, except that the auditory stimulus was fixed as “kipi.” (**B**) The duration of each visual shape was 2 s in a sequence of the stimulus presentation. One of two nonsense word sounds, “kipi” or “moma,” was presented 200 ms after the onset of each visual shape (duration: 400 ms). The duration and timing of the onset of each visual and auditory stimulus in the baseline period was identical to that in the test block.

In this study, we investigated this hypothesis using near-infrared spectroscopy (NIRS). We chose to study 11-month-old infants because multiple studies have consistently reported that infants around this age are sensitive to sound symbolism^15,17^, while the results were unstable for younger infants^16,17,29^. Another reason for this is that the participants of the previous EEG study^15^ were 11-month-old infants. While an EEG has high temporal resolution, its spatial resolution is limited. The opposite holds true for NIRS. The previous EEG study^15^ and the current NIRS study can complementarily address the neural basis of sound symbolism processing in 11-month-old infants. The word-shape pairs used in the present study were identical to those used in our previous infant study^15^ (refer to Fig. 1A for examples). The stimulus pairs, which were confirmed to elicit sound-symbolic responses in both 11-month-old infants as well as adults with various linguistic backgrounds^15^, allowed us to examine the loci of sound symbolism processing in the 11-month-old infant brain.

In each block, the infants were presented with a sequence of three spiky or round visual shapes followed by the novel word *moma* or *kipi*, which were used for Asano *et al*.’s EEG study^15^. The hemodynamic responses to the matching and the mismatching pairs were contrasted against the responses during baseline (i.e., neutral shapes followed by white noise).

## Methods

### Participants

All infants were full-term at birth and healthy at the time of the experiment. This study was approved by the Ethics Committee of Chuo University, and was conducted in accordance to the Declaration of Helsinki. We received written informed consent from the parents of all infant participants. To obtain a sufficient number of valid trials for the match and mismatch cases for analysis, the participants only heard one of the two word stimuli (i.e. the *moma* condition or the *kipi* condition. For details, see the Stimuli section). The participants included in the analyses were 22 healthy 11-month-old infants (11 in the *moma* condition and 11 infants in the *kipi* condition; 10 males and 12 females, mean age = 353 days, ranging from 336 days to 386 days). These sample sizes were determined by a power calculation on the basis of a previous fNIRS study^30^ that revealed the activation of the right STS in 7- and 8-month-old infants in response to a visual-auditory association. An additional 20 infants were excluded because they did not complete a sufficient number of trials that can be included in the analyses (fewer than three trials for either the match or mismatch presentation).

### Apparatus

Each infant sat on experimenter’s lap in an experimental booth throughout the experiment. A 21-inch color cathode ray tube (CRT) display (Sony GDM-F520) was used to present the visual stimuli. The resolution of the CRT was set at 1024 × 768 pixels with an 8-bit color mode. The display was placed in front of the infant at a distance of approximately 40 cm. The infant’s viewing behavior was monitored by a hidden video camera set beneath the CRT display. An experimenter controlled the presentation of the stimulus.

### Stimuli

Twenty spiky shapes, twenty rounded shapes, and twenty neutral shapes, which were neither spiky nor rounded, were prepared. The shapes were drawn with black lines on a white background. In each trial, infants were presented with a sequence of three different spiky or rounded shapes. Each shape was presented for 2 s; therefore, each trial lasted 6 s (Fig. 1A). Two nonsense words, *kipi* or *moma*, recorded by a Japanese female (400 ms in duration), were used as auditory stimuli respectively in two different conditions. In the *moma* condition, the auditory stimulus “moma” was presented 200 ms after the onset of a visual shapes, either a round shape (matching) or a spiky shape (mismatching) (Fig. 1B). Likewise, in the *kipi* condition, the auditory stimulus “kipi” was presented after the onset of the same shapes. These stimulus pairs were the same as in our previous infant sound symbolism study^15^ in which 11-month-old Japanese infants were found to present different EEG responses to sound-symbolically matched and mismatched word-shape pairs (see Discussion for possible issues related to the low-level acoustic features of the auditory stimuli). During the baseline period, a sequence of neutral shapes (Fig. 1A) was presented, followed by 400 ms of white noise. The duration of each visual shape and the onset timing of the auditory stimulus was identical to that in the test trials. The baseline period continued until two criteria were met: (i) the duration was >10 s; and (ii) the infant continued to look at the baseline stimuli during the last 2 s. To confirm that the shapes in the baseline stood neutral between the spiky and the round shapes, 16 adults (mean age = 26, range 20–38 SD = 4.8, 10 females) rated the associations between the nonsense words (i.e. “kipi” and “moma”) and three types of shapes. The results revealed no significant difference between the ratings of association between the “kipi” sound and the “moma” sound against the neutral shapes (*t*(15) = 1.696, *n.s*.). Furthermore, the dispersion of the ratings in the three types of shapes were similar (Levene’s test: *F*(1,30) = 0.209, *n.s*.). Therefore, any effect found in the results should not stem from the shape variabilities within each shape type.

### Procedure

Each infant was tested while sitting on an experimenter’s lap facing a CRT monitor placed 40 cm away from the chair. The infants watched the stimuli passively while brain activity was recorded. The infants were allowed to watch the stimuli as long as they were willing to do so. Their behavior was recorded on videotape during the experiment.

### The NIRS instrument

We used a Hitachi ETG-4000 system (Hitachi Medical, Chiba, Japan), which recorded NIRS from 24 channels simultaneously; 12 channels were for recording the right temporal region and 12 were for the left temporal region. The instrument generated two wavelengths of near-infrared light (695 and 830 nm) and measured the time courses of the levels of oxyhemoglobin (oxy-Hb), deoxyhemoglobin (deoxy-Hb), and total-hemoglobin (total-Hb) concentrations in each channel with a 0.1 s time resolution. We used NIRS sensor probes that were developed for infants (Hitachi Medical, infant probe 3 × 3 mode). These probes were lighter in weight and had softer skin contact than other probe types. Most of the infant participants appeared comfortable during the experiments. We used a pair of sensor probes, each of which contained nine optical fibers (3 × 3 arrays). Of the nine fibers, five were used to emit infrared light, and four were used to detect the scatter of the infrared light through the brain tissue. The optical fibers of each probe were mounted on a soft silicone holder. The emitter and detector fibers were displaced by 2 cm. Each pair of adjacent emitting and detecting fibers was assigned to a single measurement channel, which allowed for the measurement of hemodynamic changes at each of the 12 channels in each hemisphere. In each hemisphere, the centers of the probes were placed at the locations of electrodes T3 and T4 as defined by the International 10–20 electrode system. When the probes were positioned, the experimenter confirmed that the fibers were touching the infant’s scalp correctly. The NIRS system automatically evaluated if the contact was adequate to measure the emerging photons in each channel after the scattering and refraction of infrared light under the scalp.

### Data analysis of the NIRS measurements

By examining the behavior of the infants that was recorded on the videotape, we excluded from the analysis the trials during which the infant looked away from the visual stimulus or became fussy. In addition, we excluded the trials during which infants looked back at the experimenter during the preceding baseline period, and the trials with movement artifacts, which were done automatically by a computer program to detect sharp changes in the time series of the NIRS raw data. The raw oxy-Hb data from the individual channels were digitally bandpass-filtered at 0.02–1 Hz to remove longitudinal signal drifts and the noise from the NIRS system. Next, the mean concentration value of each channel within each participant was calculated by averaging the data across the trials in a time series from 2 s before trial onset to 6 s after the end of the trial, which was recorded with a time resolution of 0.1 s. Using the mean concentrations in the time series, we normalized the oxy-Hb concentration during the matching and mismatching presentation for each channel within each participant by calculating the Z-scores against the hemodynamic response during the last 2 s in the baseline. The Z-scores (*z*) were calculated by subtracting the mean concentration of the last 2 s in the baseline (μ_2_) from the concentration (μ_1_) at each time point during the stimulus presentation and then dividing this difference by the standard deviation of the concentration during the last 2 s in the baseline (σ), as follows:

$${z}=({{\rm{\mu }}}_{{\rm{1}}}-{{\rm{\mu }}}_{{\rm{2}}})/{\rm{\sigma }}{\rm{.}}$$

The difference in the signal from the last 2 s in the baseline was statistically tested. Our null hypothesis was that the brain activities in infants during the presentation of sound-symbolically matching and mismatching novel word-visual shape pairs are identical.

## Results

**Figure 2:**
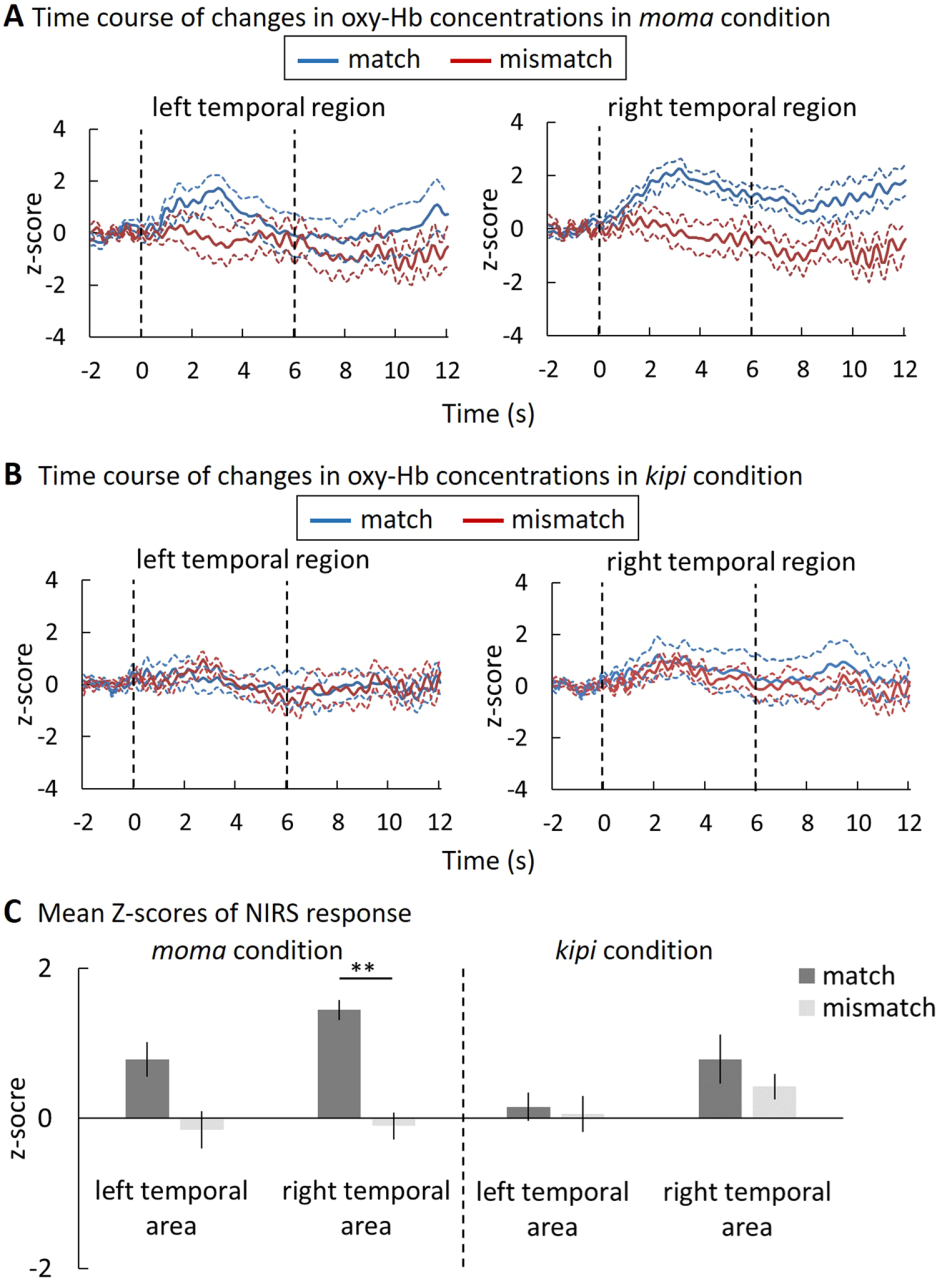
Results of the NIRS measurements in infants in the *moma* (**A**) and *kipi* condition (**B**). For both conditions, the changes in the oxy-Hb concentrations were averaged among the 11-month-old infants during the presentation of matching and mismatching pairs in the *moma* (**A**) or the *kipi* (**B**) condition. The thick blue and red lines in each panel represent the mean Z-score for the matching and mismatching trials, respectively. The broken lines represent ±1 standard error of the mean (SEM). The horizontal axis represents the time from the onset of the test stimulus (s); the vertical dashed lines at 0 and 6 s show the onset and end of the test stimulus presentation, respectively. (**C**) The mean Z-scores of the NIRS response. The left and the right panels represent the mean Z-scores of the data in infants in the *moma* and the *kipi* condition, respectively. Each bar represents the mean Z-score of the average oxy-Hb values from 0 to 6 s in the stimulus onset latency. The dark and light bars represent the results of the matching and mismatching presentation, respectively. The error bars represent ±1 SEM. The asterisks indicate statistical differences: **p < 0.01.

**Figure 3:**
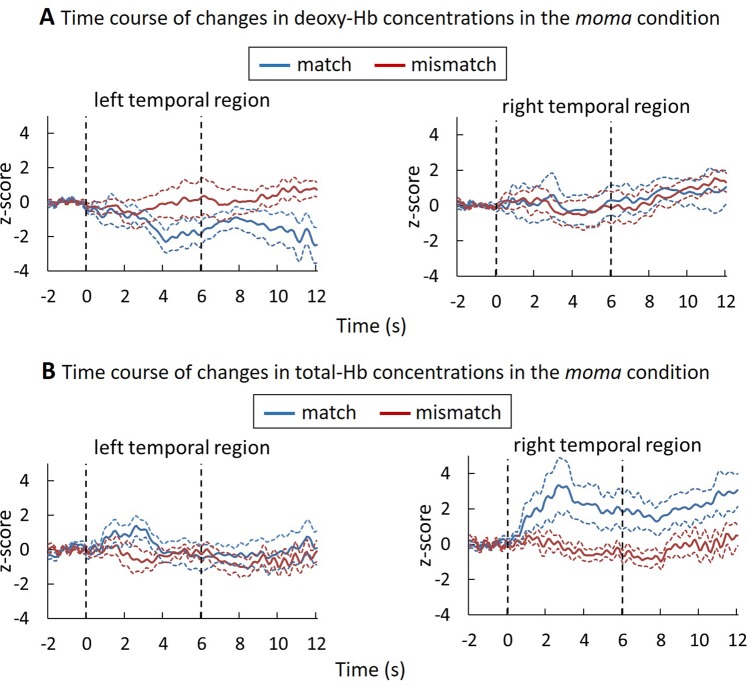
Results of the deoxy-Hb and total-Hb changes in the *moma* condition. The time courses of the changes in the deoxy-Hb (**A**) and total-Hb (**B**) concentration were averaged among the 11-month-old infants during the presentation of matching and mismatching pairs. The axes and line colors/styles are the same as in Fig. 2.

**Figure 4:**
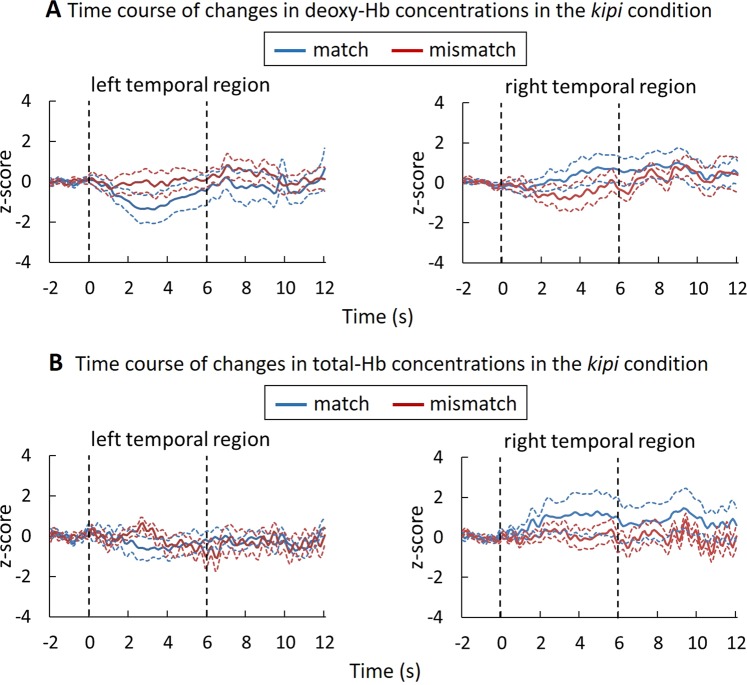
The results of the deoxy-Hb and total-Hb changes in the *kipi* condition. The time courses of the changes in the deoxy-Hb (**A**) and total-Hb (**B**) concentration were averaged among the 11-month-old infants during the presentation of matching and mismatching pairs. The axes and line colors/styles are the same as in Fig. 2.

We measured the hemodynamic responses in the temporal regions (Fig. 2). As the absolute concentration values of oxyhemoglobin (oxy-Hb) differ substantially between participants, we normalized the concentrations of oxy-Hb to the Z normalization for each channel and within each participant on the basis of the mean concentration in the time series. We first compared the responses (Z-scores), averaged across 12 channels in each the left and right temporal regions, against the baseline to assess if the hemodynamic response was modulated by the sound-symbolic correspondence between the word sound and the shape. Figure 2A,B present the time course of responses to the matching and mismatching sound-symbolical pairs. Upon visual inspection, when the sound matched the shape in the *moma* condition, the increase in the concentration of oxy-Hb in the right temporal region appeared to be much larger than in the left temporal region (Fig. 2A; results of deoxy-Hb and total-Hb changes are shown in Fig. 3). In contrast, such a difference between the hemispheres was not found when the mismatching pairs were presented. However, in the *kipi* condition, as opposed to the *moma* condition, no obvious increase in the hemodynamic response was observed either for the matching pairs or the mismatching pairs (Fig. 2B; results of deoxy-Hb and total-Hb changes are shown in Fig. 4).

To select a time window for further statistical analysis, we segmented the NIRS data into 2-second bins from stimulus onset to 10 s following stimulus presentation, and conducted two-tailed one-sample t-tests with a null hypothesis that hemodynamic responses during the experimental trials (word sound-shape match or mismatch) are not different from those during the baseline (i.e., white noise) period. The results revealed a significant increase in the oxy-Hb concentration in the 0–2 s, 2–4 s, and 4–6 s time bins [*t*(10) = 4.22, *p* < 0.01, *d* = 1.27; *t*(10) = 5.16, *p* < 0.01, *d* = 1.56; *t*(10) = 4.87, *p* < 0.01, *d* = 1.46, respectively; Bonferroni-corrected] in the *moma* condition during the presentation of the matching pairs, but not during the presentation of the mismatching pairs. In the *kipi* condition, no increase was detected in any of the time windows. Due to the significant increase in the oxy-Hb concentration observed 0 to 6 s from the time of onset, we averaged the Z-scores for this time window (mean and SD of the responses in each time bin are provided in Table 1) to test if the hemodynamic response was modulated by the sound-symbolic correspondence of the word-shape pairs.

**Table 1:** Mean (standard deviation) of oxy-Hb responses (Z-score) in each time bin in the *moma* condition.

Conditions		0 to 2 s	2 to 4 s	4 to 6 s	6 to 8 s	8 to 10 s
Match	left	0.739 (1.3)	1.200 (1.6)	0.302 (2.4)	−0.168 (2.1)	−0168 (2.1)
	right	0.904 (0.7)	1.915 (1.2)	1.480 (1.0)	1.03 (1.2)	1.030 (1.2)
mismatch	left	0.048 (1.3)	−0.158 (2.1)	−0.204 (2.1)	−0.686 (2.2)	−0.686 (2.2)
	right	0.161 (1.0)	0.077 (1.4)	−0.382 (1.7)	−0.774 (1.6)	−0.774 (1.6)

A repeated-measure ANOVA with three factors was applied to the oxy-Hb data by considering word type (*moma* vs. *kipi*), congruency (match vs. mismatch), and hemisphere (left vs. right) as the factors for comparison. This analysis revealed significant main effects of congruency [*F*(1,20) = 5.254, *p* < 0.05, *η*^2^ = 0.065] and hemisphere [*F*(1,20) = 5.671, *p* < 0.05, *η*^2^ = 0.022] and a significant two-way interaction of congruency and hemisphere [*F*(1,20) = 4.715, *p* < 0.05, *η*^2^ = 0.019]. The main effect of the word type [*F*(1,20) = 0.114, *n.s*.] and three-way interaction [*F*(1,20) = 1.954, *n.s*.] did not attain statistical significance. In order to explore the effect of word type, which we observed from the time course of responses (Fig. 2A,B), we conducted two separate repeated-measure ANOVA tests with *moma* condition and *kipi* condition. The analysis of *moma* condition yielded the observed congruency × hemisphere interaction [*F*(1,10) = 5.313, *p* < 0.05, *η*^2^ = 0.069]. This analysis also revealed a significant main effect of congruency [*F*(1,10) = 8.420, *p* < 0.05, *η*^2^ = 0.248] and a significant main effect of hemisphere [*F*(1,10) = 5.313, *p* < 0.05, *η*^2^ = 0.069]. The hemodynamic response to the sound-symbolically matched pair was stronger than to the mismatched pair, in the right temporal region [*t*(1,10) = 4.038, *p* < 0.01, *d* = 0.829], but no difference in response was observed in the left temporal region [*t*(1,10) = 1.059, *n.s*.]. In contrast, no significant main effect or interaction was found in the *kipi* condition [main effect of congruency: *F*(1,10) = 0.243, *n.s*.; main effect of hemisphere: *F*(1,10) = 2.314, *n.s*.; interaction: *F*(1,10) = 0.373, *n.s*]. Thus, consistent with our hypothesis, a reliable response to the sound-symbolically matching pair in the right temporal region was found, but the effect was limited to the “moma” sound.

To further pinpoint the cortical regions relevant to the processing of sound symbolism, we examined which NIRS channels exhibited a significant Oxy-Hb signal increase. Multiple t-test comparison revealed that channels 14, 15, 16, 20, and 23 showed a significant hemodynamic response in the *moma* condition during the presentation of the matching sound-shape pairs [ch14: *t*(10) = 4.75, *d* = 1.93; ch15: *t*(10) = 2.56, *d* = 1.06; ch16: *t*(10) = 4.00, *d* = 1.89; ch20: *t*(10) = 2.85, *d* = 1.16; ch23: *t*(10) = 3.35, *d* = 1.37; false discovery rate-adjusted *p* value < 0.05]. Again, in the *kipi* condition, no channels showed an increase from the baseline. According to the estimation of the correspondence between the channel positions in the International 10–20 EEG system and their anatomical loci^31^, these activated channels were near the right superior temporal region, which is in close topographic proximity to the key region for sound symbolism processing in adults^22^.

## Discussion

The results of this research revealed that the superior temporal region, which is known to be a cross-modal integration area, plays a key role in sound symbolism processing in 11-month-old infants, as it does in adults. Infants showed increased hemodynamic responses to sound-symbolically matched round/*moma*-type novel word-visual shape pairs in the right superior temporal area, while no such response was observed for spiky/*kiki*-type and sound-symbolically mismatched pairs. The hypothesis that sound symbolism scaffolds lexical development^3^ presupposes the ability of infants to detect the correspondence between a word sound and a referent through spontaneous cross-modal mapping prior to commencing active efforts to associate linguistic sounds to their referents. Our findings provide additional support to this hypothesis, although the results suggest that not all sound symbolism that adults may sense may be detected by prelinguistic infants^18^. More importantly, this research revealed the brain loci for sound symbolism, which is an important step toward uncovering the neural mechanism underlying sound symbolism sensitivity.

The modulation of hemodynamic responses by sound-symbolic correspondence in infants in the *moma*-sound trials was lateralized to the right temporal region. This result is consistent with the findings of the previous adult fMRI studies that identified the involvement of the right STS area in sound symbolism processing^21,22^. As it was reported that the processing of linguistic sounds is lateralized to the left temporal lobe while that of non-linguistic sounds (e.g., animal sounds, environmental sounds) is lateralized to the right temporal lobe^32,33^, the authors of the adult fMRI studies argued that sound symbolic words have properties of both linguistic symbols and non-linguistic iconic symbols and are processed correspondingly. Combined with the findings that the STS is involved in audio-visual perceptual integration in infants as well as in adults^27,28^, the right superior temporal area is predicted to be the key structure for the detection of sound-meaning correspondence in prelinguistic infants.

The attempt to draw structural analogies between the brains of infants and adults may cause concern as the former is in a state of continuous growth. In fact, previous research investigating the structural changes that occur in the brain from infancy to adulthood has suggested that maturation of the association cortex, which includes the STS area, occurs much later than the cortical area for the individual sensory modalities^34^. Despite this concern, several studies have reported that the involvement of the cortical region is analogous to the adult STS area in infant multisensory processing as well as in face processing. For example, a previous fNIRS study^28^ showed that audio-visual multisensory events triggered significant activation in the global network of the cortical areas, including the temporal areas, in 3-month-old infants. Second, functional connectivity (resting state networks) between the STS area and the visual (MT, V4), auditory (A1), and somatosensory cortices has been found to exist in the neonatal brain^35^. Thus, the neural processing of multisensory integration may involve the STS from a very early postnatal age. Additionally, previous NIRS studies have shown that activation of the right STS occurs in 7- and 8-month-old infants in response to face stimuli, similar to that typically observed in the adult brain^36–41^.

One unanticipated finding was the lack of hemodynamic change from baseline in the left temporal area in both sound-symbolically matching and mismatching conditions. Previous brain imaging (fMRI and NIRS) studies have reported that infants as young as 3- to 4-months of age recruit the left temporal areas for processing speech sounds, including pseudo-words^42–44^. Thus, it was somewhat unexpected that the presentation of novel speech sounds did not increase activation above the level observed for white noise in the left temporal region. One possible reason is that the duration time of the sound stimulus was substantially shorter in our study (400 ms) in comparison with previous studies that reported activation of the left temporal region (700–12,000 ms; 42–44). It could be that 400 ms was too short for infants of this age to invoke language processing. However, the duration of the stimulus used in our study was sufficient to invoke the response in the right STS, which strengthens the conclusion that the increased activation in the right STS reflected the perceptual cross-modal integration between vision and audition.

Another point of discussion is the finding of sound-symbolic effects for the “moma” stimulus but not for the “kipi” stimulus. Although it is difficult to specify the reason for this asymmetry, this result is strikingly consistent with the findings of a recently conducted meta-analysis on the sensitivity to sound symbolism in infants by Fort and colleagues^18^. Across the 11 studies examined, the meta-analysis found a greater sensitivity to sound symbolism for *bouba*-type pseudo-words than for *kiki*-type pseudo-words in infants and found that the sensitivity for the latter word type emerges later on. Children between 4 and 15 months of age showed a lower sensitivity to *kiki*-type pseudo-words compared to children between 25 and 28 months of age. The asymmetry between the sensitivity to the *bouba*-round and the *kiki*-spiky correspondence may arise because sound symbolic corresponding is subtler for the latter than the former. The results of the meta-analysis and the current study suggest two possibilities. The first possibility is that, infants of about 11 months of age are not sensitive to sound symbolism for *kiki*-type pseudo-words (i.e., they show only “round-*moma*” correspondence effect rather than the *bouba/kiki* effect). Perhaps the “round-*moma*” combination was easier to map than the “spiky-*kipi*” correspondence in the infants’ brain, and the latter sound symbolism needs to wait until further maturation in the brain or needs more exposure to linguistic input. Fort *et al*.^18^ suggested several possible reasons for the absence of the “spiky-*kiki*” correspondence effect, including the possibilities that, infants prefer specific acoustic and/or visual features (e.g., low-frequency *bouba*-type sounds and/or curved objects) over others (e.g., high-frequency *kiki*-type sounds/angular objects), or that infants have more experience with round objects and/or *bouba*-type words than spiky objects and /or *kiki*-type words in their direct perceptual environment^18^. The absence of the “spiky-*kiki*” effect in the present study could also have been due to the low-level acoustic features of the auditory stimuli. The sound used in this study, “kipi,” contains a vowel repetition, while “moma” contains a consonant repetition. As it has been revealed previously, infants respond differently to consonants and vowels^45^, the differences between the acoustic structures of the two auditory stimuli might have led to the asymmetric results.

Alternatively, it may be possible that the 11-month-old infants enrolled in our study were actually sensitive to sound symbolism for *kiki*-type pseudo-words, but the sensitivity was not reflected in the experimental results due to some confounding methodological factors. Temporal duration of “bouba/moma” is longer than “kiki/kipi”, and thus a stronger and temporally more sustained cross-modal mapping process should be induced by the former rather than the latter. The temporal resolution of testing methods like NIRS and preferential looking, which was used for most of the studies included in the meta-analysis^18^, might be too low to capture the transient sound-symbolic responses induced by “kiki/kipi” sounds. In fact, we observed sound-symbolic responses both in *moma*- and *kipi*-sound trials in our previous EEG study with 11-month-old infants^15^; the effect size of sound symbolism, calculated based on the mean ERP amplitudes in the sound-symbolically matched and mismatched conditions, was larger in the *kipi*-sound trials (Hedge’s *g* = 0.59) than in the *moma*-sound trials (*g* = 0.11) [see additional analyses for^18^ that is available online]. Since an EEG provides high temporal resolution, this observation is consistent with the possibility that the lack of the sound-symbolic effect was due to the particular methodological property of NIRS (as well as the preferential looking paradigm) rather than the particular sound property of the word. Of course, the last possibility is speculative, and we have no intention to argue that it is more tenable than the other two. Further research is required to verify these possibilities. However, the fact that 11 month-old infants showed sound-symbolic responses both in *moma*- and *kipi*-sound trials in our previous EEG study^15^ albeit suggests the possibility that, 11-month-old infants may have sensitivity to spiky-*kiki* type sound symbolism. It also suggests that the asymmetric results of the current study, in which we located the loci of sound symbolism processing in the 11-month-old infant brain using fNIRS, may not be only attributed to the differences between the acoustic structures of the “kipi” and “moma” sounds. To disambiguate these possibilities, in future studies, it would be beneficial to use a larger pool of novel words in which consonants and vowels are systematically combined in order to exclude the effects specific to acoustic characteristics of the auditory stimuli.

One final remark pertains to the relevance of the present result to the neonatal synesthesia theory previously proposed by some theorists^6,46^. Our result suggests that some sound-referent correspondence is processed as a spontaneous multimodal mapping, and in this sense, it is biologically based. However, it is premature to interpret the present result as evidence for or counter-evidence against the neonatal synesthesia theory. First of all, our participants were not neonates so we cannot directly speak to this theory. Furthermore, it is not clear to us whether this theory would predict that infants would detect any sound symbolism that adults would sense without learning. As noted earlier, it is possible that infants are more sensitive to particular types of sound-referent correspondences than other types prior to language learning and acquire other types of sound symbolism later through more language learning experiences. We are not certain whether this possibility counters the neonate synesthesia theory, and it is beyond the scope of the present research.

In any case, the fact that we obtained the hemodynamic changes in one type (*moma*) of sound-referent correspondence that infants have reported to be sensitive to in previous studies^18^ shows that our experimental method was valid for assessing sensitivity to sound symbolism in young infants. Although prelinguistic infants may not be sensitive to all the sound symbolism that the adults sense, what they *do* sense is processed in the right posterior temporal area, where adults process sound symbolic words. This finding is an important first step towards our understanding of the neural mechanism of sound symbolism processing, as well as understanding the ontogenesis of sound symbolism, although substantial future work is needed.
